# Participant perspectives of a telehealth trial investigating the use of telephone and text message support in obesity management: a qualitative evaluation

**DOI:** 10.1186/s12913-021-06689-6

**Published:** 2021-07-09

**Authors:** Emily Lewis, Peter Hassmén, Kate L. Pumpa

**Affiliations:** 1 University of Canberra Research Institute for Sport and Exercise, University of Canberra, Building 29, Bruce, Canberra, ACT 2617 Australia; 2 Canberra Health Services, Division of Medicine, Chronic Disease Management Unit, Obesity Management Service, Canberra, Australia; 3 Faculty of Health, Southern Cross University, Hogbin Drive, Coffs Harbour, NSW 2450 Australia; 4 Discipline of Sport and Exercise Science, Faculty of Health, University of Canberra, Canberra, Australia

**Keywords:** Obesity, Adherence, Compliance, Telehealth, mHealth, Qualitative evaluation, Focus group

## Abstract

Background: Quantitative evidence suggests that interventions involving telephone calls and text message are feasible and effective for improving lifestyle intervention adherence and clinical outcomes among adults with obesity. The aim of this article is to provide qualitative insight into the perspectives and experiences of participants who completed a telehealth trial exploring the use of telephone and text support as adjunctive tools to support a community-based obesity management program. Methods: Focus groups were conducted in order to evaluate program acceptability and overall participant perceptions of the clinical trial. Thematic content analysis was used to analyse the data, aided by the development of a thematic network. Results: The telehealth trial was well received. Participants found the telephone and text message support highly beneficial, providing encouragement, motivation and accountability via a simple and convenient mode of communication. Conclusions: These findings suggest a high degree of promise for the incorporation of telephone and text support in obesity management.

**Supplementary Information:**

The online version contains supplementary material available at 10.1186/s12913-021-06689-6.

## Background

There is a high degree of promise for the use of technology in lifestyle intervention and obesity management programs. Previous research suggests that interventions involving telephone and text message support are feasible and effective for improving lifestyle intervention adherence, weight loss, and weight loss maintenance among adults with obesity [1–7]. These modes of intervention delivery offer frequent patient-provider contact, motivation, accountability, reinforcement and support, via a cost and time effective platform.

While technology is a rapidly growing area of research in health behaviour change [3, 8], there has been insufficient research to date investigating how it can be used to increase engagement and modify adherence within lifestyle intervention programs. Our clinical trial [9] was the first study to explore the use of telephone calls and text message as adjunctive tools to support an established community-based obesity management program. This eight-month randomised controlled cross over trial was conducted in conjunction with a community-based obesity management service (OMS). Sixty-one self-managing adults with class III obesity (body mass index > 40 kg/m^2^) were randomly assigned to receive 4 months of telephone and text message support in addition to standard OMS care, or standard OMS care alone. After 4 months, the participants crossed over to the alternative sequence for four additional months. The intervention was grounded in behaviour theory, with a range of behavioural treatment strategies targeted, including goal setting, self-monitoring, motivational interviewing, problem solving, relapse prevention, stimulus control, cognitive restructuring, and self-reinforcement. Telephone calls were provided monthly throughout the intervention period, where participants were guided to set goals to work toward over the following month. The monthly telephone calls were made via Skype, scheduled at a time convenient for the participant. The information gathered during these telephone calls was then used to tailor and individualise the subsequent month of text messages. Three text messages were sent each week, aimed to remind or prompt participants to strive toward their goals, as well as foster a sense of support and accountability. This level of contact was chosen based on the level of contact seen in previous research, including a paper finding that high or low intensity text-messaging combined with group treatment equally promote weight loss maintenance in obese adults [10]. The study design involved one-way communication, so participants were unable to reply to the text messages. Instead, they were encouraged to keep notes over the month to discuss at their monthly telephone calls. One-way communication was a carefully selected component of the study design, influenced by prior research finding that low intensity single direction text message is an effective option for assisting adults involved in face-to-face obesity treatment [10]. There was not a protocol surrounding telephone call duration, with the call length varying month to month based on the number of goals being discussed, the time participants had available to talk and the general flow of conversation. The average call duration was ~ 20 min. Dietary adherence, physical activity adherence, anthropometry, self-efficacy and self-regulation were measured at baseline, 4 and 8 months. A total of 80 adults with class III obesity were approached during the recruitment phase of the trial and of these 80, 61 people posed interest in the trial and were assessed for eligibility. All 61 were deemed eligible and participated in the trial. The participants (women: *n* = 47, men: *n* = 14) had a mean age of 49 years ±12 years (25–74 years) and a mean BMI of 47.8 kg/m^2^ ± 8.4 (31.2–79.2 kg/m^2^). Baseline characteristics did not differ significantly between the two groups. Of the 61 adults who began the trial, 93% (*n* = 57) completed the 4-month outcome measures, and 84% (*n* = 51) completed the 8-month outcome measures. There was an equal drop-out rate between the two cross-over groups. The study revealed that the addition of telephone and text message support to a community-based obesity management program improved behavioural adherence and clinical outcomes when compared with standard care. Within both groups, participants achieved greater improvements in their diet, physical activity, self-efficacy and lost more weight while receiving the telephone and text support [10].

Another pertinent aspect of the program’s effectiveness lies in identifying participant perspectives and opinions of the trial. Evaluating a clinical trial using qualitative methodologies is an important step for understanding an interventions effectiveness, especially in multi-component mobile health (mHealth) programs [11, 12]. Qualitative evaluations assist researchers to identify and understand how participants view and engage with different components of mHealth interventions, with respect to the form of technology, the intensity of contact and the content delivered [13]. This information can inform intervention acceptability and possible program improvements. Similar to all qualitative research, a broad variety of methodologies may be suitable for conducting qualitative evaluations. Methods identified within previous telehealth evaluations include interviews, focus groups, surveys, retrospective reviews, feasibility studies, and mixed methodologies [14, 15]. Among the more common qualitative methods of obtaining participant perspectives are focus groups [16, 17]. Focus groups have been shown to be an effective way to obtain a diverse range of information in evaluation research [16, 18, 19], offering a useful vehicle for involving stakeholders in service development [17]. Focus group participants are encouraged to communicate openly with the research team and each other, promoting discussion around attitudes, experiences and beliefs [20]. In this way, focus groups can be a powerful tool for gaining insight into how participants perceive experiences, and may assist participants and the research team to come to mutual understanding of opinions and perspectives under discussion [21, 22]. They combine elements of both interviews and participant observation, providing an opportunity to probe participant’s experiences while also observing underlying group dynamics [16].

We enlisted focus groups to assist with the evaluation of program acceptability and to gain insight into participant experiences of the aforementioned telehealth trial. A thorough understanding of participant perspectives, engagement and satisfaction can offer insights difficult to extract using quantitative methods. For example: which components of the trial did participants find most helpful? Would they have preferred more or less frequent contact? Did they find the technology-based support convenient? Were the text messages easy to comprehend? Would additional technology features be well received? The responses to these questions may highlight possible underlying factors as to why some outcome measures showed improvement and others did not, or why some participants responded better than others. Identifying and understanding how participants engage with these modes of technology, as well as the content delivered, can inform intervention acceptability, feasibility and practicality, leading to possible program improvements.

The aim of this article was therefore to provide qualitative insight into the views, perspectives and experiences of participants who completed the clinical trial: *Adding telephone and text support improves adherence and clinical outcomes in obesity management. A randomised controlled cross over trial*.

## Methods

This study received approval from the ACT Health Human Research Ethics Committee and the University of Canberra Human Research Ethics Committee. The conduct and reporting of this research adheres to the guidelines outlined in the consolidated criteria for reporting qualitative studies (COREQ) [23]. This study has been registered with the Australian New Zealand Clinical Trials Registry (date registered: 30/03/2017), ACTRN: 12617000459325.

### Study design

A series of focus groups were conducted in order to evaluate program acceptability and overall participant perceptions of the clinical trial. The focus group session plan and topic guide are available as additional files (Additional files 1 and 2). All sixty-one participants who completed the telehealth trial were invited to participate in the focus groups following the completion of their final face-to-face outcome assessments. Participant information sheets were provided and all participants were informed verbally and in writing that the focus groups were voluntary, and if they declined to participate they would not suffer any negative consequences, such as unfair discrimination, reduction in the level of care or any other disadvantage. There was not a pre-determined number of focus groups to be conducted, rather it was planned for them to continue until all participants had been given the opportunity to attend, a pattern emerged and theoretical saturation had been achieved [17]. Participants were offered remuneration for fuel at a rate of $0.66 per kilometre travelled. The groups were held within a community-based education group room that was familiar to all participants. The literature suggested six to ten participants per focus group was an optimum number to gain a variety of perspectives in a manageable and orderly fashion [17, 24]. The groups ran for approximately 1 h. There was pre-existing acquaintance amongst some of the focus group members, however a clear benefit of pre-existing acquaintance had been found in prior research [25], and was therefore not purposefully avoided.

### Data collection and analysis

Two female research team members facilitated the focus groups. Dr. E.L. (PhD) was the primary investigator leading the telehealth trial and had an established relationship with the participants. Associate Professor K.P. (PhD) was a co-investigator on the telehealth trial and had no established relationship with the participants. Dr. E.L. (PhD) is an Accredited Practicing Dietitian and an Accredited Exercise Physiologist, specialising in obesity management. She has a diploma in qualitative research methods and design. Associate Professor K.P. is an Accredited Practicing Sports Dietitian and an Accredited Exercise Physiologist who convenes the Bachelor of Exercise Physiology and Rehabilitation degree within the Discipline of Exercise and Sports Science at The University of Canberra. Participants were aware of the focus group and overall research objectives. The researcher’s reasons for completing the study were also discussed prior to commencement of the focus groups. There wasn’t anyone else present at the focus groups besides the participants and the researchers. All groups were audio-recorded, after verbal and written consent was obtained. Participants were informed of their right to request at any stage for the audio recording to be temporarily paused. The audio recordings were transcribed verbatim, de-identified to protect participants’ privacy. Transcripts were not returned to participants for comment. Along with the supplementary session notes, the transcribed focus groups were organised and analysed using thematic content analysis [16, 26]. This analysis was aided by the development of thematic networks; web-like illustrations that summarise the main themes constituting a piece of text [26]. Thematic networks constitute (i) basic themes; simple premises characteristic of the data, (ii) organising themes; categories of basic themes grouped together to summarise principles, and (iii) global theme(s); a super-ordinate theme encapsulating the principle meanings within a text as a whole [26]. Initially a coding framework was formulated, allowing the data to be re-organised, refined and reinterpreted under the guide of the study’s key objectives. This was completed by the study’s primary investigator (E.L.). Relationships and themes within the data were then identified, organised within thematic networks and described in detail. This was completed collaboratively amongst the three study investigators (E.L., K.P., P.H.). A reflexive approach was inherent in our analysis and interpretation of the findings. No software was used to manage the data. Participants did not provide feedback on the findings.

## Results

**Fig. 1:**
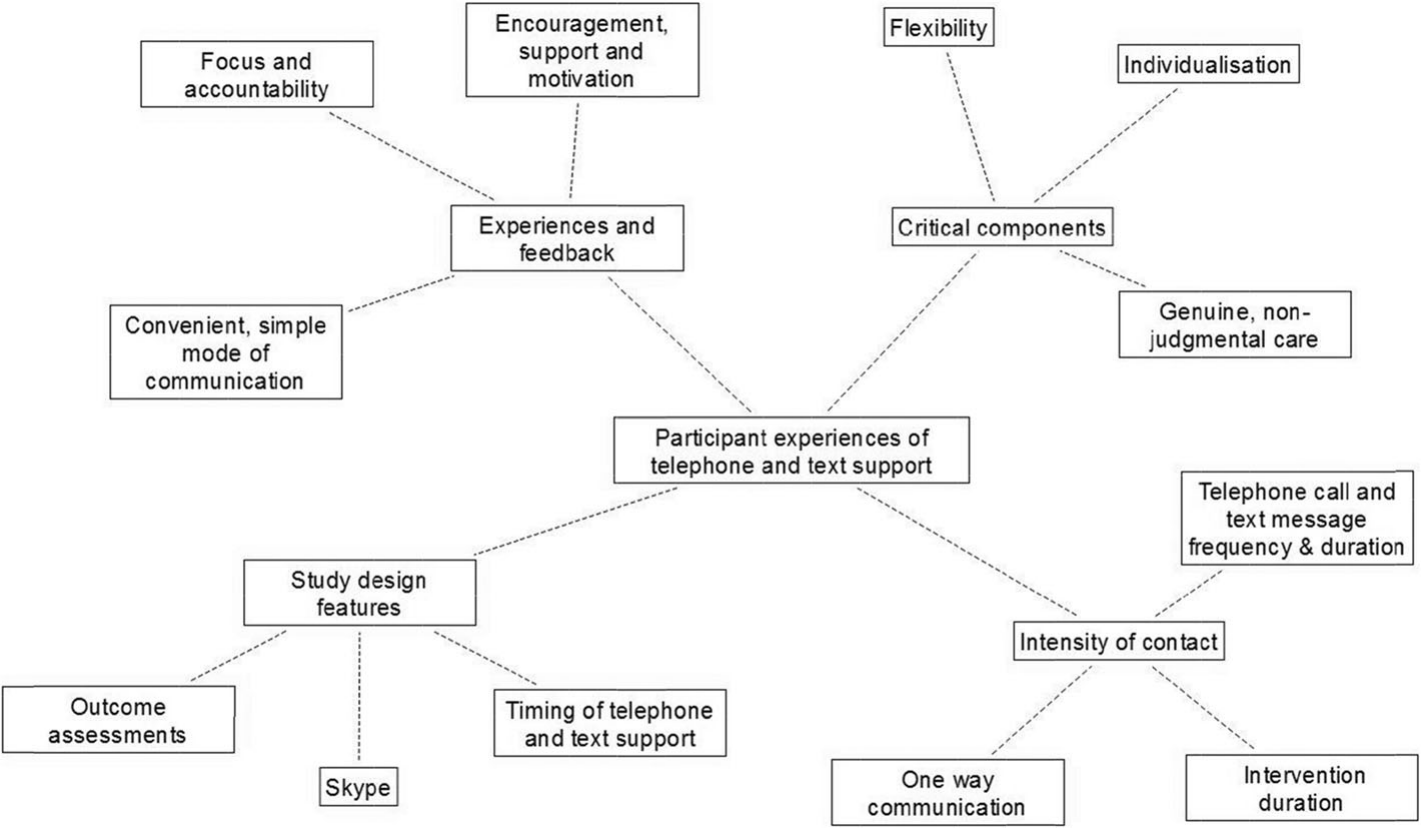
Thematic network of participant perspectives of a telehealth trial investigating the use of telephone and text support in obesity management

Three focus groups were required for a pattern to emerge and theoretical saturation to be achieved. No participant took up the offer of fuel remuneration. Of the 61 adults invited to participate, 33 posed interest, with a total of 15 participants attending focus groups. Characteristics of those who completed the focus groups and telehealth trial are summarised in Table 1. There were no significant differences in age, sex distribution, baseline weight, waist circumference or body mass index. Those who agreed to participate within the focus groups achieved higher percentage weight losses from baseline to 8 months than the average seen within the telehealth trial. The thematic network is presented in Fig. 1 and described in detail in the following sections.

**Table 1:** Baseline characteristics of participants who attended the focus groups and completed the telehealth trial

	Participants completing focus groups (*n* = 15)	Participants completing telehealth trial (*n* = 61)	
Variable	Mean ± SD, n, %	Mean ± SD, %, n	*P*-value
Age	55 ± 12 years	49 years ±12.6	0.63
Female: Male ratio	12:3	47:14	0.47
Baseline weight	127.1 kg	132.0 kg	0.42
Baseline waist circumference	126.5 cm	127.1 cm	0.20
Baseline body mass index	47.0 kg/m^2^	47.8 kg/m^2^	0.45
Percentage weight loss	5.4%	3.0%	0.56

### Experiences and feedback

#### Focus and accountability

The telephone calls and text messages helped participants maintain focus on their health and lifestyle goals. Phrases such as: *‘keeping my mind on it’; ‘staying on track’; ‘keeping me focused’; ‘stopping me getting distracted’;* and *‘keeping me accountable’* were common throughout the focus groups. Many said they need assistance to stay on track and can easily lose focus on their goals without regular reminders and support.

*‘The texts were very good for me because often I’ve started diets and things before trying to control my weight, but I find that something happens and my weight goes up and I can’t cope with it. Keeping my mind on it for the first few weeks was very important for me. I can’t be trusted entirely by myself to stay focused, but you kept me accountable.’ – Participant 4*

Accountability was a prominent topic of discussion, with participants agreeing that having somebody to keep them accountable and monitor their progress on a regular basis was invaluable.

#### Encouragement, support and motivation

There was unanimous agreement that the telephone calls and text messages offered much needed support, encouragement and motivation. Many participants felt they were not receiving enough support through the OMS alone, so the telephone calls and text messages offered the contact and encouragement they were craving.

*‘I found it positive, because I felt as if there could be a lot more support. So getting that text or phone call was a real positive feeling for me. To think, oh I haven’t been forgotten- to actually have some support.’ – Participant 15*

A few participants joked that the text messages seemed to arrive at exactly the right moment, for example when they were making a decision about what to order for dinner or whether to head to the gym. They found the text messages empowering and gave them the motivation to take action toward their health and lifestyle goals.

#### Convenient, simple mode of communication

Participants found the mode of communication convenient, simple and effective. They enjoyed not having to take time off from work or the need to travel, with many saying the time saving factor of technology-based communication was very helpful and worked well with their busy lifestyles.

*‘I loved the telephone call because I didn’t have to take time off work. I didn’t have to come all the way over to Belconnen to talk to you. And quite often my telephone call with you was while I was going for a walk, so I could find that I was fitting more into my day.’ – Participant 2*

*‘I think you are saving a lot of time. You can just see on your phone there is a message there, and even if you don’t have time to read it you can sit down some time later when its quieter and read it.’ – Participant 6*

They also enjoyed the ability to re-read previous text messages at times when they felt they needed the extra encouragement. Many said they referred back to particularly poignant text messages regularly for boosts of motivation. A few participants remarked that in long face-to-face appointments they struggle to absorb all of the information. This pressure to take everything in was removed with the technology-based support, because the text messages acted as mini summaries of the telephone call, delivered every few days. In that way the text messages weren’t only offering support, but also ongoing reminders and prompters.

*‘Often with an hour face to face appointment you miss heaps. And I take notes but I still miss stuff. Whereas this is like a little telegram, it just sits there and you can go back to it and refer to ones from months ago.’ – Participant 12*

Participants were asked if other forms of technological support, such as email, video chat, website or phone application would be of interest. There was widespread consensus that other forms of technological support were not warranted and would not be well received. Participants liked the simplicity and ease of the telephone calls and text messages, with many saying they do not have enough technological literacy to attempt other forms of communication.

*‘I liked the simplicity of it. I am only just getting used to emails- texts are enough for me! I’m a bit of a technophobe, so I would have hated it.’ – Participant 10*

*‘I don’t work in front of a computer so it would have been harder and inconvenient for me to have had to email and things like that. I know you can do it off your phone but it’s still painful.’ – Participant 7*

One participant broached the topic of push vs pull technology [27], saying that push technology was a safer option for keeping people motivated.

*‘In terms of maintaining motivation, I think you’re better off with a push mechanism, which is like the text. Because I couldn’t be trusted I don’t think to go and pull the information from an app or a website.’ – Participant 1*

This concept of push vs pull technology was explained to the group and most participants agreed that push technology was a better option for them. A few participants discussed that often websites have such a wealth of information that it can be overwhelming, whereas a text message is just a short snippet that can be digested easily at their own convenience.

### Critical components

#### Flexibility

The telephone calls were scheduled at times convenient for the participant, commonly during lunch-breaks, in the evenings or on weekends. This flexibility was possible for the primary investigator, and ensured a conversation that was thorough, unhurried and purposeful. The call was generally scheduled a few days in advance, and participants were encouraged to spend time thinking about their goals and progress in the lead-up so that the calls could be efficient and productive. There was widespread agreement that having flexibility with scheduling the telephone calls was invaluable, with many stating that they simply couldn’t have participated if the calls had to be conducted at set times.

*‘It would have been impossible for me. Because of my roster and shift work and I can’t have my mobile phone at work so it would have been impossible.’ – Participant 1*

*‘In a work environment, being in and out of meetings all day, with so much in my diary, I wouldn’t have been able to stick with a set time, or it would have been very difficult.’*

*– Participant 8*

#### Individualisation

There was a strong and resounding view that individually tailoring the text messages was a critical feature of the intervention. Participants appreciated the attention to detail and thoughtfulness that went into individualising their text messages.

*‘I could tell there was a lot of thought that had gone into the content and that it was perfectly tailored for me. There was real care and concern in it. I really appreciated the time and the thought that went into what it was that I needed to hear in a way that I was going to be able to receive it as well.’ – Participant 5*

Participants felt strongly that the text messages would not have held the same impact if they came from a standard template and weren’t individually tailored.

*‘I would have resented it if it was on a standard template. Because we are intelligent people, we could read that on the internet. We know that stuff, the issue isn’t that we don’t know what we should be doing; the issue is that it needs to be personalised. And if I read a text that I knew was the same as everyone else’s, I would have just deleted it. Yeah I do feel very strongly about that part of it.’ – Participant 1*

*‘I really appreciated the tailored nature of them and I think I would have just ignored a series of pro forma messages.’ – Participant 2*

Not only was the content of the text messages tailored to the individual, but also the day of the week and time that they were sent was carefully determined based on the participants’ lifestyle and goals. For example, those who were attempting to go to the gym after work would be sent a text in the late afternoon to encourage them to get there. Likewise, people who aimed to exercise in the morning would often get a text the evening before to remind them to set their alarm and put their exercise clothes out. Others would receive inspiration on the weekend to get outside and exercise or to organise their groceries and meals for the week. Participants were asked if they enjoyed the text messages arriving at different times throughout the week, and if they would have minded the texts being sent at set times, for example 3 pm Monday, Wednesday, Friday. They agreed that having the text messages individualised to their lifestyle and goals and sent at varying times across the week and month was appreciated.

*‘It was more connected to what you were thinking about doing. So I’d get a text on say a Friday night saying did you think about taking the kids to the farmers markets or something on the weekend and I was like, “oh! that’s a good idea”, and then I’m thinking about what I’m going to do on the weekend. So it was really well connected to your life I guess. I think if it came at routine times it might lose its power.’ – Participant 8*

*‘I really appreciated the tailored messages. I think once you tailored them because I was travelling, and they came at the right time, in the right time zone.’ – Participant 2*

This aspect of the intervention may seem labour-intensive, however the texts were generally written in advance and scheduled for sending throughout the week or even the month. Choosing the time and day of the week for a particular text to be sent was therefore easily chosen while writing the text. This seemingly small feature of the study design proved to be one of the most appreciated components.

#### Genuine, non-judgmental care

Ensuring the text messages offered inspiration and encouragement without pressure or intrusion was critical. We know that people’s receptiveness to text message varies significantly depending on the wording, information and tone [28]. It was therefore paramount to consider factors such as age, education level, sex, stage of change, culture and socio-economic status when constructing the texts. The monthly telephone calls were included within the intervention for this reason, to allow the clinician/researcher to get to know the participant and to keep in touch with changes in their lives or their goals on a regular basis. The focus group discussions revealed that participants found the text messages encouraging and supportive, without feeling any pressure or judgment.

*‘There was no pressure or lecturing there, it was so non-judgmental.’ – Participant 10*

*‘Agreed. The other thing that I appreciated was that if I was struggling with something I felt the freedom and acceptance to be able to say that, rather than: “she’s expecting this answer so I better give this answer”. I felt that there was a real ability to be able to say, actually no I’m struggling with this bit or, yeah, gosh I’m glad you reminded me of that. And I never felt condemned in that, I always felt encouraged and reminded of what I had done well so far and then, well this is a new moment now, how do I move forward from here, so that’s something I really appreciated.’ – Participant 5*

The focus groups highlighted the importance of injecting care and compassion into the technology-based support. While many said that clinical factors such as the expertise or professional background of the clinician, or the specific information offered within the text message was important, everyone agreed that personal attributes such as being genuine, caring and respectful was even more critical.

*‘There was real care and concern in it. You were authentic and very genuine- I could tell that you really did care. It wasn’t just that this is my job and this is my thing and this is what I’m doing. It was something that you really... I felt very cared for in the process of it, it wasn’t just clinical. And the right balance of empowering me but also where I felt I could just be free with where I was at.’ – Participant 9*

### Intensity of contact

#### Telephone call and text message frequency and duration

Participants were asked about the intensity of contact, including the frequency of text messages; the frequency of telephone calls; and the duration of the telephone calls.

- ➢ Text message frequency

The majority of participants felt that three text messages per week was the right amount, with everyone agreeing that more frequent contact was not necessary.

*‘It was enough. With all due respect, I’m not being negative there, but it would not have had the impact or the meaning if it was an everyday thing. If it became a daily text it wouldn’t have been worth as much. It would lose the impact.’ – Participant 10*

One participant felt that three text messages per week was appropriate at the beginning of the intervention, but became too frequent toward the end and could have been tapered off.

*‘I found that initially it was good, but towards the end I was getting three or four texts per week, it felt a little bit much, if you know what I mean. Because I am reasonably motivated anyway and I felt that maybe one or two max per week would have been ok toward the end.’ – Participant 3*

Participants liked the idea of being given choices regarding text frequency in the latter half of the intervention, with some saying they would stick with three and others thinking they might reduce it to one or two.

- ➢ Telephone call frequency

The majority of participants felt that a monthly telephone call was ample, with many stating that the month between calls flew by quickly.

*‘I just remember commenting every time we spoke that “oh my god has a month gone past already!?” I felt like it didn’t take a month to get to the next call and I wouldn’t think that any more frequent phone calls would have been any more valuable. It would have just added impost to the process where I have to step out and take a phone call every two weeks rather than month. And even when it was monthly I was just amazed at how frequent the month came around. So I don’t think frequency was an issue at all, monthly was enough.’ – Participant 8*

*‘It was close enough to be continual but not far enough to be, “oh god, what did we talk about last month” sort of thing. I think it was perfect timing.’ – Participant 11*

There were a few participants who would have appreciated fortnightly calls.

*‘Probably every fortnight would have been helpful for me. Because it’s just a juggling act for me, and I put myself last. So having a reminder “hey, you are still important and keep focused on what you’re doing as well”; that’s where I found it really helpful.’ – Participant 15*

- ➢ Telephone call duration

There wasn’t a protocol surrounding telephone call duration, with the call length varying month to month based on the number of goals being discussed, the time participants had available to talk and the general flow of conversation. The average call duration was ~ 20 min, and all participants agreed this duration was appropriate.

*‘Appropriate to our needs, if it was a short one it was appropriate, if it was a long one we had things to discuss and you made the time to do that. I never ended the calling feeling like “what was that?” It was always clear and I knew exactly what I had to do.’ – Participant 13*

#### Duration of intervention

The telephone and text support was offered for a duration of 4 months. The majority of participants would have liked the support to be offered for longer; agreeing that the additional support would continue to be beneficial if the duration was extended. Many thought that 6 to 12 months would be ideal, whereas others simply felt that ongoing support was necessary.

*‘I think ongoing would be good. To give you the confidence to keep going and not slow down.’ – Participant 14*

Many participants commented that their progress slowed when the telephone and text support came to an end.

*‘As a larger person with a lot to lose, I loved the four months, but I really noticed it once it had stopped.’ – Participant 5*

*‘I loved it, and I needed it, and I felt that when it stopped, in the four months that it wasn’t there, that my progress decreased markedly.’ – Participant 2*

A popular suggestion was for the support to be tapered off gradually. For example, rather than the monthly phone calls and three text messages per week to end suddenly, the telephone calls could be spaced to bimonthly and the text messages could reduce to one or two per week. This tapering could continue until either a set time, or the individual and clinician felt that the added support was no longer necessary. This may be a way to feasibly extend the duration of the technology-based support within a community environment.

*‘One suggestion- I had been getting regular messages and then nothing, so I would have liked a taper, say going to one message a week and then a message a month, just “hey I’m still here, how are you going?”... So there was a taper that continued at the end of the four months. If we had that sort of a weaning process that would be a nice way to finish.’ – Participant 12*

#### One-way communication

The study design involved one-way communication, so participants were unable to reply to the text messages. There were mixed views amongst participants when asked whether they would have liked the ability to respond to text messages. The majority of participants said they didn’t feel the need to respond, with one participant even saying she was relieved that she couldn’t text back.

*‘In a way I was glad I didn’t have to text back. It was a relief; there then wasn’t pressure to text back all the time. I didn’t feel rude for not texting back.’*– Participant 12.

A few participants said there were times when they wished they could have written back.

*‘I wish that I could reply sometimes. Sometimes I wanted to say “yeah, I’m smashing this”, and I wanted to be able to tell you that. I would never have wanted to reply and say I’d done nothing that week, but when I was doing really well I wanted to tell you.’*

*– Participant 2*

*‘Sometimes I thought that as well, I think because there was that … championing, and that goal setting process with you, when we reached those goals and actually did them, it would have been nice to be able to give you that feedback.’ – Participant 9*

### Study design features

#### Timing of telephone and text support

The trial involved a cross over design, with one group receiving the telephone and text message support in addition to standard care initially, followed by standard care alone, while the other group received standard care alone first, followed by the additional telephone and text message support. The optimal timing of receiving the telephone and text support was therefore under investigation. The trial found there were similar improvements between the two groups for all of the outcome variables between baseline and 8 months, therefore there was no quantitative evidence to indicate an optimal timing of the intervention. However, an equally pertinent question remained: what did participants think about their group allocation and when do they think is the ideal time to be offered the technology-based support? On the whole, participants said they would prefer to receive the additional support a few months into their journey with the OMS. Participants who received the telephone calls and text messages straight away said they would have preferred a delay. They found there was already a lot of support on offer at the OMS in the initial months and having the telephone calls and text messages at this time was not necessary and at times overwhelming. They felt that the face-to-face contact and general support offered by the OMS slowed down after a few months, so when the telephone calls and text messages came to an end they were left somewhat *‘on their own’*. This group felt that the intervention would have had more impact if it were offered mid-way through their time at the OMS when the clinical contact, and often motivation, was beginning to wane.

*‘I was in the first group where I had support straight up, and it was quite overwhelming. As supportive and great as it was, just with all of the weekly visits and the sessions that we had to attend to be a part of the OMS, as well as being in the first group, I felt like I lived here a bit. So I think maybe in the middle or towards the end would be good so that you’ve got yourself into some sort of routine and you know what to expect and you know what you’ve got to do and then magic Emily is going to come and kick you in the butt if you don’t continue it. When the motivation is starting to die a little.’ – Participant 7*

Participants who received the telephone calls and text messages in the second sequence of the trial agreed that this was the better group to be in.

*‘I was in the second group and I think it worked quite well. You’ve got the first four months where you’re learning about changing your diet and exercise, so you’re changing your lifestyle and focusing on that. And then seeing you later was good timing and worked quite well - I think it might have gotten lost at the beginning.’ – Participant 14*

#### Skype

The monthly telephone calls were made via Skype. The research team was interested to see if there were any issues encountered from the use of Skype. Nobody had any issues with the mode of communication, with participants saying they hadn’t even realised that the calls weren’t made from a landline phone.

#### Outcome assessments

Overall, only minor comments were highlighted regarding the outcome assessments. Participants were asked if they found the four monthly outcome assessments burdensome, or if there were any issues with the assessment tools. Participants did not find the assessments to be onerous, saying they were reasonably quick and simple. A few participants commented that the questionnaires were ambiguous, and they found some of the questions to be contradictory and difficult to answer. There were mixed views around the use of the activity monitor (SenseWear Pro3 Armband Mini), worn around the upper arm for a one-week period. A few participants enjoyed wearing the armband and receiving the feedback on their physical activity and energy expenditure. One participant found the armband irritated their skin. A few wished they could have worn the armband in water as pool-based exercise was their primary form of exercise and they were disappointed the activity monitor was not able to capture this data. These views confirmed the research teams impression that a water-resistant activity monitor validated for this type of research, once commercially available, would better reflect the improvements in physical activity adherence.

## Discussion

The aim of this study was to provide qualitative insight into the views, perspectives and experiences of participants who completed the clinical trial: *Adding telephone and text support improves adherence and clinical outcomes in obesity management. A randomised controlled cross over trial*. This qualitative evaluation reveals that the telehealth trial was well received, with participants reporting positive experiences and outcomes. Participants found the telephone calls and text messages highly beneficial, providing encouragement, support, motivation and accountability via a simple and convenient mode of communication. They did not feel that other modes of technology, such as websites, email, phone application or video-chat would be sought after, preferring the simplicity of telephone and text. Genuine, non-judgmental care; individually tailoring the text messages; and flexibility regarding scheduling of the telephone calls were all considered to be crucial aspects of the intervention. Participants appreciated the individualisation of the text messages in regard to both content and time of delivery, noting the power and impact may have been lost if a standard template was used. A monthly telephone call and three text messages per week was considered to be an appropriate amount of contact, with only a few participants suggesting more frequent communication. The majority of participants would have liked the telephone and text support to be offered for a longer duration, ideally ongoing. In regard to the optimal timing of the intervention, participants preferred to receive the telephone and text support a few months into the OMS program. This delay allows time to complete the initial education groups run by the OMS, and to evenly distribute the degree of support on offer throughout their OMS journey. There were no issues encountered through the use of Skype, and only minor comments regarding the outcome assessments and assessment tools used within the clinical trial.

Participants in this qualitative evaluation expressed their desire for frequent patient-provider contact and regular, ongoing support. Previous research in obesity management has shown that intensive patient-provider contact is an important component of a programs popularity and effectiveness [29, 30]. This approach is typically seen within academic lifestyle intervention programs, but often deemed too burdensome and expensive to be sustainable in community environments. Intensive patient-provider contact requires considerable resources including time, money and the availability of multiple health professionals with expertise in the behavioural treatment of obesity. One potential way to alleviate pressure, resources and the demand on face-to-face contact is through the use of technology [31]. The use of telephone calls and text message offers a unique opportunity for obesity management programs, and a range of similar health care services, to provide frequent patient-provider contact, prompting and reminders via a cost and time-effective platform, while reducing the reliance on face-to-face treatment. The telehealth trial revealed that telephone and text support improves adherence and clinical outcomes in obesity management, and this qualitative evaluation offered strong validation that participants believe telephone and text support is an invaluable addition to a community-based obesity management program.

The findings of the current study are in line with previous qualitative evaluations investigating participant perceptions of telehealth and mHealth interventions [13–15, 32–34]. These qualitative papers reveal that telehealth trials are generally well received, with participant’s reporting positive experiences and offering promising feedback. Previous evaluations have also found participants have a clear preference for telephone calls over other forms of technological support (e.g., email, video chat, website or phone application). This preference appears to be a result of the simplicity, ease and individualisation that telephone calls are able to offer. A qualitative evaluation looking at postpartum women’s perspectives of receiving nutrition and physical activity support via video consultation noted convenience and improvements in knowledge and confidence as primary benefits [15]. They concluded that offering tailored support via video consultation was a feasible and acceptable method of delivering care, and a viable alternative to in-person care. In another example, a process evaluation was completed to investigate participant perceptions of and engagement with TXT2BFIT: a multi-component mHealth randomised controlled trial to prevent weight gain in young adults [13]. This paper reported positive feedback overall, with the personalised coaching calls being the most valued component of the intervention, offering motivation and accountability. Participants viewed the other components of the intervention, such as phone applications and websites, to act as supplements to the coaching calls, as opposed to core components. The participants expressed their preference for personalised intervention, which is why the telephone coaching calls were deemed to be the preferred component of the intervention. The text messages were enjoyed and offered helpful reminders, although participants would have preferred for the texts to be individualised [13]. Participants preferred behaviour-based messages as opposed to knowledge-based messages, and were more likely to implement the behaviour if it was related to a goal discussed in the coaching call. This strengthens the view that personalised support is integral, and is in line with evidence suggesting that self-monitoring, goal checking, prompting and behavioural cues are important for behaviour maintenance [35, 36]. Participants of this study also suggested a more gradual decline in contact frequency would have been helpful, which is line with the tapering of support suggested by participants of the current study. Neither of the above examples involved concurrent face-to-face intervention, and participants within both of these examples would have preferred more frequent contact and support.

Focus groups were the chosen methodology to gain participant perspectives and experiences within this qualitative evaluation. The focus groups proved to be a successful tool, yielding rich information, feedback, opinions and experiences. The insights gained through the focus groups were not obtained using quantitative methods and would have been otherwise unexplored without this qualitative evaluation. Participants appreciated the opportunity to offer their feedback and further contribute to the body of research being conducted at the OMS. The research team also found the focus groups to be highly beneficial, allowing a years worth of anecdotal feedback to be formally collated, analysed and presented. Gaining interest in the focus groups was not a difficult task, with a large number of participants posing interest in attending. Unfortunately, only a small proportion of those who wanted to attend were able to, as the focus groups needed to be conducted during the working day due to room availability and health centre open hours. This was interesting, supporting the notion that for many individual’s telehealth is a convenient and preferable option of health care consumerism, whether that be for traditional health care or for research purposes. In hindsight, mixed methodologies including surveys and telephone interviews may have been a more valuable and robust option, allowing everyone who wished to offer their feedback the opportunity to do so. Especially given the qualitative evaluation was seeking feedback from people who had agreed to participate in a telehealth trial, continuing to offer online forms of communication may have resulted in higher participation rates.

There are a number of limitations of the study to consider. Participants who agreed to participate in the focus groups achieved higher percentage weight losses than the average seen within the telehealth trial. Their experiences may therefore be biased toward a positive experience. The sample of participants may have been small, however data saturation was achieved quickly. The primary investigator leading the telehealth trial co-facilitated the focus groups. It is difficult to ascertain whether this pre-existing relationship affected the responses provided and the overall outcomes. Likewise, the primary investigator of the telehealth trial was heavily involved in analysing the transcribed focus group data, including the creation of the coding framework and the organisation of themes within the thematic network. There is therefore a risk of researcher bias due to the primary investigator’s key role within both the telehealth trial and the qualitative evaluation. One strategy used to address this potential bias was having two co-investigators with no prior relationship with the participants involved in the interpretation and analysis of the data. A reflexive approach was also inherent in our data analysis, factoring in the influence of researcher bias on the resulting findings. The qualitative evaluation may have been strengthened if further strategies were implemented to account for this potential bias. The focus groups included a mix of participants and were not specifically designed by subgroups (i.e., age, gender, sex etc). Furthermore, all participant voices were considered together during the analysis and interpretation of the focus group data. Accounting for differences in how different types of participants felt about the intervention may have proven interesting and offered a richer insight into the participant perspectives. Flexibility around scheduling the telephone calls was deemed a critical component of the trial. If this type of technology-based support were to be implemented, services would need to balance this need for flexibility with what is feasible and practical for service delivery.

The telehealth trial and this qualitative evaluation have been successful in demonstrating the benefits of using telephone calls and text messaging in obesity management. However, it is important to highlight that this research has investigated the use of telehealth as an adjunctive tool alongside traditional face-to-face intervention delivery. In this context, for individuals who do not have access to a mobile phone, or the technological literacy required to benefit from text message support, face-to-face service delivery remains. Telehealth offers additional patient-provider contact, accountability and prompting, however standard health care is still on offer for those who do not wish to partake. This is important in the context of digital equity. While evidence suggests that the vast majority of the population has a cell phone [3, 37], it is crucial that health care is still provided to those who do not (or for those who are simply not interested in receiving health care via this mode of delivery). That being said, mobile phones have had a considerable impact on health care availability around the world, especially in developing countries [3]. The use of mobile technology to promote health and prevent disease is growing at a rapid rate [3, 38]. Communication by mobile phone is generally less expensive than landline telephones or standard internet and offers the ability to provide health care to vulnerable groups, rural communities and resource-poor settings in which people may not have easy access to health care professionals or expensive technologies [3, 33, 37, 38]. There is strong potential in text messaging specifically as it is available on nearly every mobile phone, has widespread use, is relatively low cost, is a convenient form of communication and is widely applicable to a wide array of health conditions and behaviour change interventions [3, 38].

While telehealth has long been touted as a useful tool in the health service model, the pandemic has led to a rapid change in the way services can be delivered. Consumer expectations have shifted and there is now demand for convenience in a safe and socially distant manner, supported by readily available existing technology. Telehealth is therefore likely to become embedded as an adjunct to regular service delivery models, and more prevalent within health care research.

## Conclusion

The perspectives and experiences of participants who completed a telehealth trial investigating the use of telephone and text message support in obesity management were overwhelmingly positive. These findings suggest a high degree of promise for the incorporation of telephone and text support as adjunctive tools to support community-based obesity management programs. The positive experiences suggest that this type of additional support is an effective, efficient and convenient means of improving obesity management service delivery. Given the rapidly increasing demand for these services, it represents an opportunity to provide the additional support required, within finite resource constraints. This knowledge is a powerful driver for obesity management services looking to implement some form of telehealth within their current model of care.

## Supplementary Information

**Additional file 1.** Focus group session plan.

**Additional file 2.** Focus group topic guide.

## Data Availability

The datasets analysed during the current study are available from the corresponding author on reasonable request.

## Abbreviations

OMS: Obesity Management Service
E.L.: Emily Lewis (study author)
K.P.: Kate Pumpa (study author)
P.H.: Peter Hassmén (study author)
