# Supplementary material for: Participant perspectives of a telehealth trial investigating the use of telephone and text message support in obesity management: a qualitative evaluation
Source: BMC Health Serv Res. 2021 Jul 9;21:675. doi: 10.1186/s12913-021-06689-6 (PMC8268488; doi:10.1186/s12913-021-06689-6)
Supplement: Supplementary file 1 — Additional file 1. Focus group session plan. [file 12913_2021_6689_MOESM1_ESM.docx]

Focus group session plan

Welcome participants

Introduce facilitators

Refresh housekeeping – location of restrooms, water etc.

Introduce topic

Introduce purpose, benefits and general focus group procedure

Establish group agreement that information disclosed within the group will remain in the room, and that mutual respect for participants and facilitators be upheld at all times.

Explain that the focus group will be audio-recorded, however participants can request for the recording to be temporarily paused at any stage.

Turn on audio recording

State the focus group date, time, number of people present and facilitators present

Use focus group topic guide as a guide, however allow the focus group to unfold through participant discussion and conversation

At completion of the focus group thank participants for their attendance
