# Supplementary material for: Participant perspectives of a telehealth trial investigating the use of telephone and text message support in obesity management: a qualitative evaluation
Source: BMC Health Serv Res. 2021 Jul 9;21:675. doi: 10.1186/s12913-021-06689-6 (PMC8268488; doi:10.1186/s12913-021-06689-6)
Supplement: Supplementary file 2 — Additional file 2. Focus group topic guide. [file 12913_2021_6689_MOESM2_ESM.docx]

Focus group topic guide

1. Impression of telephone calls
   - Frequency of calls
   - Duration of calls
   - Mode of telephone call (was there difficulty hearing using Skype?)
2. Impression of text messages
   - Frequency of texts
   - Content of texts – was individualisation of texts received well or could the texts be standardised?
   - Language used/ tone of texts
   - Using participants first name and signing off with investigators name
3. Would it be preferable to have the technology-based support delivered by the participants OMS case manager? Or was it helpful to have this offered by an external provider?
4. Was the technology aspect of the intervention convenient/ helpful as opposed to face-to-face appointments?
5. Would additional technology features be well received e.g. telephone application, website etc.
6. Overall impression of intervention
   - 4-month duration of technology support – too long or short?
   - For those who received the technology-based support in the first half of the intervention, was this early support helpful or, with hindsight, would they have preferred to have received this half-way through their time at OMS? Alternatively, for those who received the technology-based support in the second half of the intervention, was this later support helpful or would they have preferred to receive it earlier to assist them to get started?
   - Would they recommend the use of telephone calls and text message as a means of motivation and support within the OMS?
